# Microglia Specific Drug Targeting Using Natural Products for the Regulation of Redox Imbalance in Neurodegeneration

**DOI:** 10.3389/fphar.2021.654489

**Published:** 2021-04-13

**Authors:** Shashank Kumar Maurya, Neetu Bhattacharya, Suman Mishra, Amit Bhattacharya, Pratibha Banerjee, Sabyasachi Senapati, Rajnikant Mishra

**Affiliations:** ^1^ Department of Zoology, Ramjas College, University of Delhi, Delhi, India; ^2^ Department of Zoology, Dyal Singh College, University of Delhi, Delhi, India; ^3^ Department of Molecular Medicine and Biotechnology, SGPGI, Lucknow, India; ^4^ Immunogenomics Laboratory, Department of Human Genetics & Molecular Medicine, Central University of Punjab, Bathinda, India; ^5^ Biochemistry and Molecular Biology Laboratory, Department of Zoology, Banaras Hindu University, Varanasi, India

**Keywords:** Brain, Neuroinflamamation, Natural product, Microglia, Neurodegeneration

## Abstract

Microglia, a type of innate immune cell of the brain, regulates neurogenesis, immunological surveillance, redox imbalance, cognitive and behavioral changes under normal and pathological conditions like Alzheimer’s, Parkinson’s, Multiple sclerosis and traumatic brain injury. Microglia produces a wide variety of cytokines to maintain homeostasis. It also participates in synaptic pruning and regulation of neurons overproduction by phagocytosis of neural precursor cells. The phenotypes of microglia are regulated by the local microenvironment of neurons and astrocytes via interaction with both soluble and membrane-bound mediators. In case of neuron degeneration as observed in acute or chronic neurodegenerative diseases, microglia gets released from the inhibitory effect of neurons and astrocytes, showing activated phenotype either of its dual function. Microglia shows neuroprotective effect by secreting growths factors to heal neurons and clears cell debris through phagocytosis in case of a moderate stimulus. But the same microglia starts releasing pro-inflammatory cytokines like TNF-α, IFN-γ, reactive oxygen species (ROS), and nitric oxide (NO), increasing neuroinflammation and redox imbalance in the brain under chronic signals. Therefore, pharmacological targeting of microglia would be a promising strategy in the regulation of neuroinflammation, redox imbalance and oxidative stress in neurodegenerative diseases. Some studies present potentials of natural products like curcumin, resveratrol, cannabidiol, ginsenosides, flavonoids and sulforaphane to suppress activation of microglia. These natural products have also been proposed as effective therapeutics to regulate the progression of neurodegenerative diseases. The present review article intends to explain the molecular mechanisms and functions of microglia and molecular dynamics of microglia specific genes and proteins like Iba1 and Tmem119 in neurodegeneration. The possible interventions by curcumin, resveratrol, cannabidiol, ginsenosides, flavonoids and sulforaphane on microglia specific protein Iba1 suggest possibility of natural products mediated regulation of microglia phenotypes and its functions to control redox imbalance and neuroinflammation in management of Alzheimer’s, Parkinson’s and Multiple Sclerosis for microglia-mediated therapeutics.

## Introduction

The brain is one of the immune-privileged organs whose mechanisms of the immune response are poorly understood. However, immune-responsive lymphocytes, microglia, and transcription factors have been observed in the brain. Pathological conditions in the brain have been observed associated with increased oxidative stress which leads to redox imbalance. The microglia responds to pathogens and injury. They accumulate in the region of inflammation or deterioration. It produces cytokines to maintain brain homeostasis, during development, adulthood and aging (Bitzer-Quintero and Gonzalez-Burgos, 2012; London et al., 2013; Burda and Sofroniew, 2014). They serve to communicate between the central nervous system and the peripheral immune system (Perry and Teeling, 2013). The microglia gets primed to release pro-inflammatory cytokines during chronic systemic inflammation, neuronal dysfunction, viral or bacterial infection, healthy aging, and dementia (Perry and Teeling, 2013). During postnatal brain development, microglia regulate the over-production of neurons via phagocytosing neural precursor cells, shape synaptic fields through synaptic pruning (Kim and Joh, 2006; Paolicelli et al., 2011; Li et al., 2012; London et al., 2013; Nayak et al., 2014). Studies also suggest that microglia support the laminar structure of the cortex, outgrowth of dopaminergic axons, and neurite formation (Pont-Lezica et al., 2011; Squarzoni et al., 2014).

In an adult healthy brain, microglia remain in a “resting” or “quiescent” state with a ramified morphology (Hernangomez et al., 2014; Andreasson et al., 2016; Hristovska and Pascual, 2016; Reemst et al., 2016; Arcuri et al., 2017). The microglia of aged organisms show activated state, morphological changes, alterations in telomere length, increased expression of MHC-II, and CD11b. They limit neurogenesis during aging and induce neuroinflammatory reactivity in astrocytes, reduce synapse and pre-synaptic function. Since aged astrocytes are induced by microglia and become neuroinflammatory, the targeting of astrocytes may protect synapses in aging-related diseases (Boisvert et al., 2018). The malfunctioning of microglia cause redox imbalance and increased oxidative stress leading to the recruitment of lymphocytes from blood to damaged sites (London et al., 2013).

## Biology of Microglia

Microglia was identified as a small population of phagocytic, migratory cells of mesodermal origin by (del Rio-Hortega, 1932). In 1939, del Rio-Hortega coined the term “microglial cell.” The two statements from del Rio-Hortega while describing microglia raised the controversy over microglia origin. In one statement, he proposed that microglia may arise from mesodermal cells of the innermost layer of meninges i.e., pia mater. In another statement, he proposed that microglia may of myeloid origin because of phenotypic similarities in morphology and phagocytic activity like mononuclear cells of the blood. Despite some controversies (Ginhoux and Prinz, 2015) on the mesodermal or ectodermal origin of microglia, several investigators presented data in support of the mesodermal origin of microglia. Through a series of experiments, it was further revealed that unique embryonic precursors, the yolk sac macrophages, are precursors of microglia. They are not found in the bone marrow. They reached the brain rudiment and appear to persist there into adulthood (Ginhoux and Prinz, 2015).

### Microglia, Redox Imbalance and Neurodegeneration

Microglia cells serve as innate immune cells in the brain and accumulate in regions of degeneration and produce a wide variety of cytokines to maintain homeostasis. The local microenvironment of neurons and astrocytes also affects phenotypes of microglia by interaction with both soluble and membrane-bound mediators (Wohleb, 2016). When neurons degenerate, either due to acute or chronic neurodegenerative diseases, the microglia get released from neurons and show activated phenotype (Perry and Teeling, 2013; Chen and Trapp, 2016). They exhibit dual functions. When the signal is short and moderate, microglia shows a neuro-protective phenotype to clear cell debris by phagocytosis and release growth factors for the healing of neurons. In acute and chronic signals, microglia release pro-inflammatory cytokines like TNF-α, IFN-γ, reactive oxygen species (ROS), nitric oxide (NO), all of which endanger neuronal damage. The malfunctioning of microglia results in the recruitment of lymphocytes from blood to damage sites, which further maintain brain homeostasis (London et al., 2013; ElAli and Rivest, 2016; Donat et al., 2017; Skaper et al., 2018). The microglia-mediated secretion pro-inflammatory cytokines, ROS and NO helps in restoring brain homeostasis as described. However, same redox imbalance or elevated oxidative stress also acts on microglia and affects its function. Increased inflammatory cytokines also induces microglia to secrete high levels of glutamate which in turn suppresses astrocytes glutamate transporters essential for maintaining extracellular physiological glutamate levels leads to excitotoxicity mediated neuronal damage. Gap junctions and hemichannels also play critical role in communication between different cells and are involved in development and progression of various neurological diseases. Literature suggest that activated microglia release excess of glutamate via Cx32 hemichannels. Excessive release of glutamate and proinflammatory cytokines from microglia also disrupt gap junctional communication and increased activity of hemichannels in astrocytes, adversely affect brain homeostasis and increase the severity of disease (Orellana et al., 2009; Takeuchi and Suzumura, 2014). Therefore, identification of microglia specific genes and its targeting with natural products may help in bringing back microglia to resting state, restoring its immunological surveillance function and in regulation of redox balance in the brain to mitigate disease conditions.

### Signature Genes of Microglia

In activated microglia, Ionized binding protein1 (Iba1), a 17 kDa calcium-binding EF-hand protein, participates in actin-bundling, membrane ruffling, cell migration, and phagocytosis (Ito et al., 1998; Ito et al., 2001; Ohsawa et al., 2004). The Iba1-positive cells have small nuclei, scanty cytoplasm, and branched processes (Ito et al., 1998). In facial nerve axotomy, ischemia, and several other brain diseases, microglia show activated phenotype with up-regulated expression of Iba1 (Ito et al., 1998; Ito et al., 2001). The Iba1 immunostaining was also considered useful to study the pathophysiological role of activated microglia (Ito et al., 2001). It gets up-regulated by the pro-inflammatory cytokine IFN-γ and forms complex with L-fimbrin and co-localizes with F-actin and small GTPase Rac participates in membrane ruffling and phagocytosis activity in activated microglia, whereas Iba1 mutant mice completely lack membrane ruffling and phagocytosis (Oshawa et al., 2000; Kanazawa et al., 2002). In the hippocampus, the morphology of Iba1 positive microglial cells exhibits small somata with thin tortious and ramified processes. Iba1 has been shown to localize in the cytoplasm and nucleus of microglia (Jinno and Kosaka, 2008). In the dentate gyrus, Iba1 positive microglia were seen in the inner molecular layer, sub-granular zone and also found in the border between granule cell layer and molecular layer. The Iba1 has also been described as more suitable markers than trans-membrane and plasma membrane specific markers for studies on the complex organization and structural analysis of microglia (Korzhevskii and Kirik, 2016). Genome-wide transcriptome and epigenome studies have shown that microglia is different from other tissue macrophages and glial cells. It was also found that *Cx3cr1, Trem2, Tyrobp, Cd33, Sall1, Tmem119, Siglec-H, Iba1*, and *P2ry12* specifically expressed in mouse microglia (Chiu et al., 2013; Galatro et al., 2017). Recently, another microglia specific marker Tmem119 has been identified as a marker of mature microglia in the brain (Bennett et al., 2016; Satoh et al., 2016; Bohnert et al., 2020) and Siglec-H expression specific to activated microglia where it cause phagocytosis of glioma cells of brain tumor (Kopatz et al., 2013; Konishi et al., 2017) and Olfml3 as a new Tgf-β1/Smad2 target gene in microglia (Neidert et al., 2018) has been reported. However, detailed studies on targeting these identified genes to understand microglia function in the regulation of oxidative stress and neurodegeneration are still elusive.

## Disease Associated Microglia, Modulations of Microglia-Specific Genes and Their Interactions in Neurodegeneration

Microglia act as the first responders to infection and inflammation in the CNS followed by migration of the peripheral macrophages into the CNS during neuroinflammation. Activation of microglia by intensive acute or chronic stress cause loss of essential function of microglia and they start showing more of deleterious effect, which in turn cause immune compromised and alteration in CNS protection (London et al., 2013). Understanding the disease-associated-microglia (DAM), its heterogeneity and key regulators, and emerging “microgliome” will help to distinguish transcriptionally distinct and neurodegeneration-specific microglial profiles for clinical research and drug discovery (Sousa et al., 2017). The innate immune receptor and its adaptor protein, such as triggering receptor expressed on myeloid cells 2 (TREM2) and DNAX-activating protein of 12 kDa (DAP12), has been associated with a range of CNS disease phenotypes (Painter et al., 2015). Impaired signalling in disease pathogenesis is caused by mutations in TREM2-DAP12 pathway leading to altered microglial survival and proliferation which in turn affect phagocytosis, cytokine production, and immune responses (Konishi and Kiyama, 2018). In the TREM2-DAP12 signaling, the TREM2 signal via DAP12 receptor activates the protein tyrosine kinase ERK (ERK1/2), regulating actin cytoskeleton polarization and cytoskeleton organization and stimulates microglial phagocytosis. TREM2 also upregulates the expression of the CCR7 chemokine receptor (Mecca et al., 2018). Secondly, TREM2 interaction with DAP12 promotes microglial survival by activating the Wnt/β-catenin signalling pathway. TREM2-DAP12 interaction activates PI3K/Akt signaling, leading to blockage of GSK3β and stabilizing β-catenin that moves into the nucleus, thereby triggering pro-mitotic and anti-apoptotic genes. TREM2-DAP12 signaling activates PI3K/Akt pathway through the regulation of NF-κB (inflammatory genes) and inhibition of RAF (MAPK signaling) causing TLRs signaling inhibition (Zheng et al., 2017; Mecca et al., 2018). Gene ontology analyses coupled with bioinformatics approaches (such as weighted co-expression network analysis, WGCNA) and confirmation in AD mouse models (adult WT and 5xFAD mice) have revealed distinct pro-inflammatory and anti-inflammatory phenotypes within DAM (Rangaraju et al., 2018). Results proposed that the pro-inflammatory DAM was characterized by higher expression of CD44, CD45, and Kv1.3 channels and are controlled by NFkB, Stat1, and RelA signaling pathways, while the anti-inflammatory DAM profile was characterized by CXCR4 expression and regulated by LXRα/β (Rangaraju et al., 2018). Further, the findings in human AD suggested an increase in the expression of pro-inflammatory DAM proteins at pre-clinical stages which showed a positive link with tau and neurofibrillary tangle pathology (Rangaraju et al., 2018). A recent report highlighted the potential therapeutic targets in modulating microglial functions for Alzheimer’s disease (AD) as NLRP3 inflammasome activation (VRAC, P2X7, NLRP3), complement production and signalling (C1, C1q, C3, CR1, Factor B, Factor D, and Properdin), and TREM2/DAP12 signalling (TREM2, SHIP-1, PLCγ2, and Apolipoprotein E) (Nizami et al., 2019). Alterations in TREM receptors (TREM-1/2) are emerging as key components associated with inflammatory bowel disease (IBD) and neurodegenerative disorders spreading inflammation through the gut-brain-axis namely microbiota dysbiosis, leaky gut, and inflammation (Natale et al., 2019). Over the years, the novel molecular and functional signature for brain-resident microglia has been proposed. Neurodegeneration models have proposed cell markers like P2ry12, Fcrls, Siglec-H, Olfml3, and Tmem119 (Chiu et al., 2013; Butovsky et al., 2014; Bennett et al., 2016), and several of them need to be validated further. An anatomically complete atlas of the adult human brain transcriptome critically analyzed transmembrane protein 119 (TMEM119) in bulk human brain tissue data from the Allen Brain Institute (Hawrylycz et al., 2012). The Tmem119 is a stable and robustly expressed microglial marker of brain-derived microglia in both mice and humans (Bennett et al., 2016). The cell-type expression profiling tool (CellMapper) identified 30 additional genes along with TMEM119 i.e., *ACY3, ADAM28, ADORA3, ALOX5AP, C1QB, C3, CD33, CD84, CIITA, CPED1, CSF2RA, DHRS9, FCER1G, FYB, GPR34, HPGDS, IGSF6, LAPTM5, LY86, P2RY13, RASAL3, SASH3, SELPLG, SPN, SUCNR1, SUSD3, SYK, TBXAS1, TLR7*, and *TREM2* (Bonham Luke et al., 2019). Interestingly, scientists found that microglial module genes are strongly expressed in numerous healthy human brain regions known to be vulnerable in AD. Recent studies using weighted gene co-expression network analysis (WGCNA) strongly characterized a gene network that relates to both aging and neurodegenerative diseases (Mukherjee et al., 2019). Since research on the human brain transcriptome is restricted to post-mortem tissue analyses, thus mouse genetic experimentation mouse is now widely characterized as the model organism of choice for studying the diseases of humans and brain aging, as they share 99% of their genes with humans (Rosenthal and Brown, 2007). Thus, the discovery of similarly preserved networks and cell type signature genes in animal models will help to get mechanistic insights and analyze differential expression in Alzheimer’s disease, Parkinson’s disease, and related disorders.

## Molecular Mechanism of Key Microglial Proteins and Their Impact on Neurodegenerative Diseases

**FIGURE 1:**
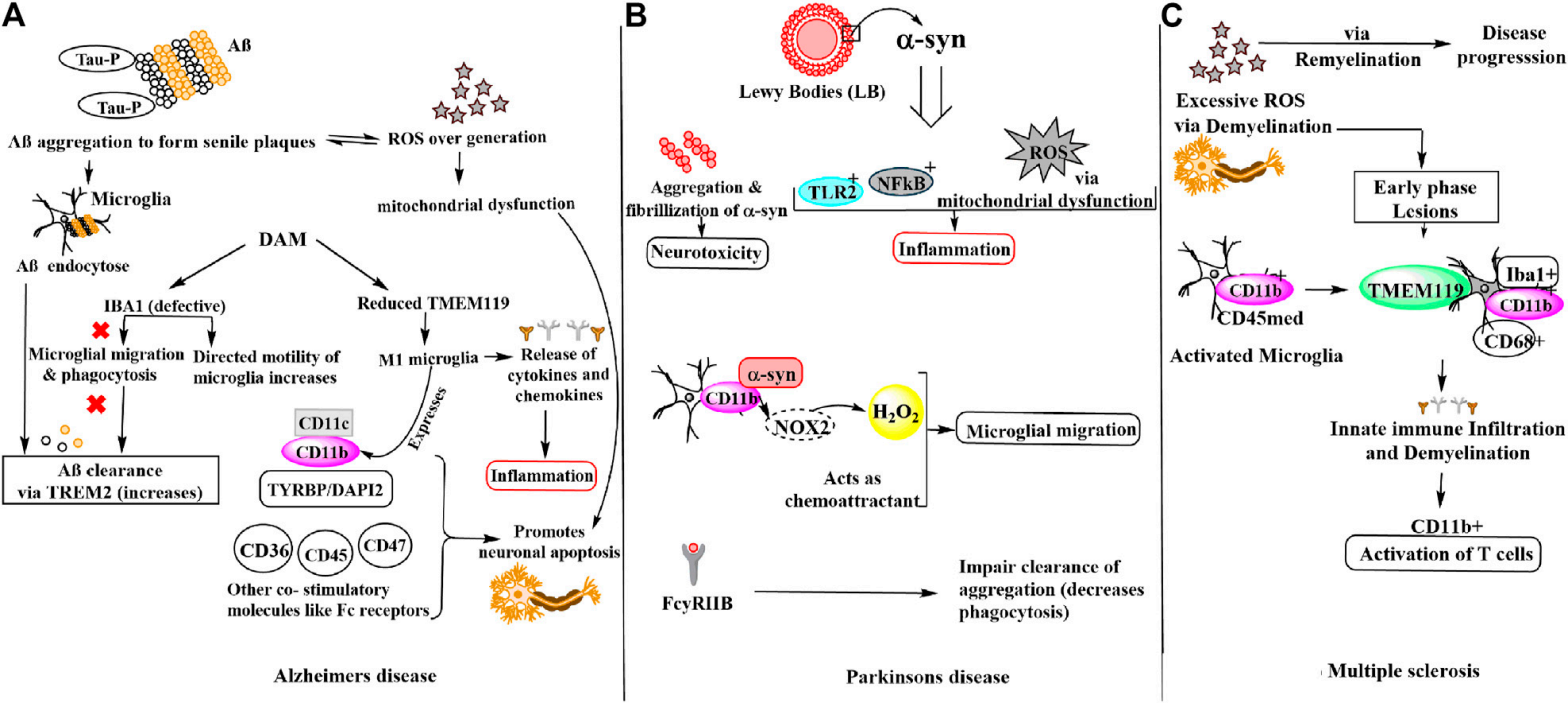
Schematic representation of microglial involvement in disease pathogenesis of **(A)** Alzheimer’s disease **(B)** Parkinson’s disease **(C)** Multiple sclerosis.

Neurodegenerative diseases are a complex group of disorders characterized by progressive decline in structure and function of central and/or peripheral nervous system. The most common neurodegenerative diseases are Alzheimer’s disease, Parkinson’s disease and Multiple Sclerosis. These diseases lead to loss of neurons and thus affect the brain homeostasis. The disease pathogenesis very often caused by factors like oxidative stress, neuroinflammation, altered clearance mechanism, deposition of proteins like amyloid-beta, alpha-synuclein etc., malfunctioning of brain-specific immune cells (microglia), disturbed blood-brain barrier, and infiltration of lymphocytes. Many mechanisms and key genes have been identified which play a central role in neurodegenerative disease where involvement of microglia specific proteins showed the importance in regulating healthy immune system of brain to combat the effects of neurodegeneration (Figure 1). In this review diseases like Alzheimer’s, Parkinson’s and Multiple Sclerosis disease were discussed to highlight the microglial proteins which have a key role in brain and neuronal homeostasis.

### Alzheimer’s Disease

Alzheimer’s disease (AD) is a progressive neurodegenerative disorder affecting majorly the elderly population. Specific molecular signatures of AD include extracellular amyloid plaques deposits of amyloid-beta (Aβ) peptide, neurofibrillary tangles of tau (microtubule-binding protein), dystrophic neurites, loss of neurons and synapses and fibrous glia (Murphy and LeVine, 2010; Hansen et al., 2018). The tau protein hyper-phosphorylation observed at Ser202/Thr205, Thr212, Ser214, Thr217, Ser262, and Ser422 amino acid residues in AD due to oxidative stress results in neurofibrillary tangles via self-assembly of paired helical filaments admixed with straight filaments (Miao et al., 2019). Further, the aggregates of AD-P (AD-hyperphosphorylated) tau are seen in dystrophic neuritis around the Aβ plaque (Vickers et al., 2016). Tau pathology has also been found to be linked with reactive microglia, astrocytes and increased levels of pro-inflammatory molecules including cytokines and complement proteins (Vogels et al., 2019). It is noteworthy that in per mole of tau protein, 2–3 mol of phosphate is present in normal condition. However, during anesthesia and hypothermia a transient rise is observed while in AD pathology, the phosphate concentration increases drastically i.e., >6 mol/1 mol of tau (Iqbal et al., 2010; Simic et al., 2016; Miao et al., 2019). Another hallmark of AD pathology is the oxidative stress due to redox imbalance caused by overproduction of ROS. Excess of ROS or free radicals lead to nucleic oxidation, protein oxidation and/or lipid peroxidation, metal homeostasis disturbance and mitochondrial dysfunction. Notably, there is a vicious cycle that modulates ROS by Aβ secretion or vice versa. It is also reported that generation of Aβ via APP processing leads to enhanced ROS generation (Moneim 2015; Wojsiat et al., 2018). Aβ40 and Aβ42 monomers produced from APP processing by β-secretases, γ-secretases, oligomerize and aggregate into senile plaques that lead to activation of microglia. Microglia then endocytose Aβ aggregates contributing toward their clearance via TREM2 surface receptors, simultaneously triggering an innate immune response against the aggregation (Tiwari et al., 2019). Earlier, this microglia reaction was thought to be concomitant and triggered by amyloid deposits and dystrophic neurites in AD. However, recent GWAS (genome-wide association studies) have identified that the majority of AD risk loci are located within or near to the genes that are highly and sometimes uniquely expressed in microglia. Thus, microglia being critically involved in the early steps of the disease and identified as important potential therapeutic targets (Hemonnot et al., 2019). Notably, in both physiological and disease states the microglia expresses Iba1 which participates in both baselines and directed motility. In AD pathogenesis, microglia shifts from baseline to directed-motility, involving proteins including Iba1, P2ry12, Cfl1, and Coro1a. Aβ immunotherapy suggested to result in increased microglial baseline motility (suggested by increased *IBA1* and *P2RY12* expression, which are considered “homeostatic” microglial markers), potentially due to decreased Aβ and/or tau, but without restoration of the physiological status of microglia (Franco-Bocanegra et al., 2019). To identify microglia in humans and experimental models *IBA1* has been widely used as a marker as it is distributed along the cell body and processes. However, there is experimental evidence that microglial motility is impaired in AD, for instance in a post-mortem study this was observed by the changes seen in the interactions of motility-related proteins with Aβ as well as with each other. Iba1 is crucial for the formation of actin bundles (Ohsawa et al., 2000), which in turn help in the formation of lamellipodia, filopodia, and membrane ruffles essential for microglial migration and phagocytosis, important for Aβ clearance (Ohsawa et al., 2000; Zotova et al., 2013). In disease-associated microglia (DAM) state, expression of some homeostatic gene, like TMEM119 (transmembrane protein 119) and other genes (e.g., CX3CR1 and P2RY12) is reduced but their major role in AD is unknown (Hansen et al., 2018). Although another molecule CD11b (αM integrin), along with the increased expression of *APOE, TREM2, CD68, CLEC7a*, and *ITGAX (CD11c)* referred to as DAM, microglial neurodegenerative phenotype (MGnD), or activated response microglia (ARM) are also observed under neurodegenerative conditions (Keren-Shaul et al., 2017; Krasemann et al., 2017; Sala Frigerio et al., 2019). Remarkably in AD, M1 (proinflammatory) microglia express MHC-II integrins (CD11b, CD11c) and other co-stimulatory (CD36, CD45, CD47) and Fc receptors. Thus, it is found that in microglia, CD11b is required with TYROBP/DAP-12 to control the production of microglial superoxide ions that promotes neuronal apoptosis during brain development. Additionally, M1 microglia produces pro-inflammatory cytokines and chemokines, ROS and NOS. While, M2 microglia produce anti-inflammatory cytokines (IL-10, TGF-β), growth factors (IGF1, FGF, CSF1), and neurotrophic growth factors (nerve-derived growth factor, brain-derived neurotrophic factor (Bdnf), neurotrophins, glial cell-derived neurotrophic factor (Gdnf), which downregulate, protect, or repair in response to oxidative stress and inflammation (Sarlus and Heneka, 2017).

### Parkinson’s Disease

Parkinson’s disease (PD) is the second most common neurodegenerative disorder after AD with a sharp increase in incidence after 60 years (de Lau and Breteler, 2006). PD is characterized by four cardinal signs (resting tremor, bradykinesia, rigidity, and postural instability). Notably, the hallmark of PD is the presence of Lewy bodies (LBs) whose primary structural component is filamentous α-synuclein (α-syn) (Eriksen et al., 2005; Kouli et al., 2018) and the degeneration of dopaminergic neurons in the substantia nigra pars compacta responsible for the motor impairments (Moore et al., 2005). α-syn was found to increase ROS levels in dopaminergic cells. Thus, it was reported that oxidative damage could be modulated by α-syn perhaps via modulation of mitochondrial function because mutant α-syn over-expression leads to significant enhanced sensitivity of dopaminergic neurons to mitochondrial toxins including mitochondrial processing peptidases (MPP)^+^ and 6-hydroxydopamine, ensuing an augmented protein carbonylation and lipid peroxidation (Chinta and Andersen 2008). Although, identification of *SNCA* genes that encodes α-syn and many other genetic variants, susceptibility for PD plays a crucial role in the disease pathogenesis. Interestingly, reactive microglia and neuroinflammation have been well known in fibrillary formation in PD (McGeer et al., 1988; Polymeropoulos et al., 1997). Microglia and the innate immune system are essential for synaptic pruning to impart changes to the neural world around them and suggested their contribution to both neurological and psychiatric illnesses (Stevens et al., 2007; Hong et al., 2016; Sekar et al., 2016; Vainchtein et al., 2018). In the same manner, microglia-derived inflammation might stimulate astrocytes to adopt a neurotoxic role or to lose neurotrophic or synaptoptrophic functionality (Liddelow et al., 2017). However, the microglial response to excess or mutant α-syn is still under investigation. The innate immune surface receptors of microglia including toll-like receptor 2 (TLR2) get activated by extracellular α-syn oligomers that function as damage-associated molecular patterns (DAMPs) (Kim et al., 2013; Kam et al., 2020). Under physiological conditions, α-syn regulates the trafficking of synaptic vesicles and the formation of the SNARE complex in the presynaptic terminals, but in pathologic states, α-syn undergoes aggregation and fibrillization that results in neurotoxicity in PD (Burre et al., 2015). Microglia targets highly active neurons by sensing and modulating neuronal activity in the brain (York et al., 2018). The neurons release α-syn in CSF and extracellular space, as well as conditioned media of neurons in a calcium-dependent manner (Emmanouilidou et al., 2010) and crucial for the so-called spreading of α-syn and dependent on neuronal firing (Emmanouilidou et al., 2016; Yamada and Iwatsubo, 2018). Furthermore, microglial migration is being directed by Α-syn as a chemo-attractant. Interaction CD11b with α-syn and subsequent NOX2 activation lead to increase H_2_O_2_ (hydrogen peroxide), which can also act as a final direct signal for migration (Wang et al., 2015a). This is evident with the *post-mortem* study of PD patients where the activated microglia is found in close contact with neurons presenting Α-syn pathological accumulation (Croisier et al., 2005). However, it should be noted that microglia can and will encounter α-syn by the trogocytosis of neuronal structures as well (Weinhard et al., 2018). α-syn interacts with microglial surface receptors including Fc gamma receptor IIB (FcγRIIB) to reduce microglial phagocytosis, which in turn could impair the clearance of aggregated species or other parenchymal debris (Choi et al., 2015). Inflammatory microglial response activated by fibrillary α-syn via NF-κB pathway (Yun et al., 2018). Microglial uptake of α -syn fibrils is regulated by Fyn kinase and class B scavenger receptor CD36 which was genetically linked to PD as per recent GWAS (Nalls et al., 2019; Panicker et al., 2019). Microglia releases IL-1β through a signalling mechanism that led to NLRP3 inflammasome priming and activation. Thus, aggregated α-syn induces pro-inflammatory microglial behaviors via both classic innate immune receptors and interactions with intracellular signaling cascades. (Kam et al., 2020). During high levels of inflammation by chemokines such as CX3C ligand 1 (CX3CL1) produced by neurons or C-C motif chemokine ligand 2 (CCL2) and CXC ligand 10 (CXCL10) produced by microglia, astrocytes, and other inflammatory cells, peripheral NK cells are recruited to the CNS. Other important inhibitory receptors found on Natural Killer (NK) cells are NKG2A for Qa-1b and CD244 for CD48 in mice and CD85 for HLA-A, CD94 for HLA-E in humans. CD48 found on the surface of lymphocytes and other immune cells, endothelial cells, and dendritic cells is a member of the CD2 subfamily of immunoglobulin-like receptors which include signalling lymphocyte activation molecules and functions in activation and differentiation pathways of these cells. CD48 does not have a transmembrane domain but is held at the cell surface by a GPI anchor via a C-terminal domain which may be cleaved to yield a soluble form of the receptor. Microglia-NK cell interaction exerts protective function which can induce decreased expression of the MHC class I molecule Qa1 on activated microglia, which in turn triggers NK cell-mediated cytotoxicity towards hyperactive microglia. As microglia are known to become aberrantly activated in the presence of sustained α-syn burden, targeting microglial activation states by suppressing their deleterious pro-inflammatory neurotoxicity may be a valid therapeutic approach for PD treatment. Furthermore, NK cell-deficient animals displayed increased inflammation in the CNS, as shown through glial fibrillary acidic protein (GFAP) and Iba1 (Mihara et al., 2008).

### Multiple Sclerosis

Another chronic inflammatory neurodegenerative disease of the central nervous system (CNS) is multiple sclerosis (MS) which is characterized by demyelination and neuroaxonal damage that leads to the formation of lesions throughout the central nervous system (CNS). Pathological conditions of MS also include generation of excessive ROS, reactive nitrosative species (RNS) responsible for oxidative stress and redox imbalance. ROS plays dual role in causing axonal damage that subsequently leads to neurodegeneration in MS either by affecting demyelination causing apoptosis of oligodendrocytes or remyelination process by oligodendrocyte precursor cells maturation disruption. ROS can also activate NF-kB which alleviates the expression of TNFα and many other genes involved in MS pathogenesis (French et al., 2009; Cai and Xiao, 2016; Cunniffe and Coles, 2021). Gray matter (GM) demonstrated a low number of activated microglia and little infiltration of lymphocytes compared to white matter (WM) lesions (Deng and Sriram, 2005). The early stage of MS is characterized by relapses and the later stage, by progressive disability. Microglia and macrophages play a crucial role in the disease course by actively initiating immune infiltration and the demyelination cascade during the early phase of the disease while promoting remyelination involved in disease progression in later stages. A controversial distinction of microglia from monocyte-derived macrophages depends on expression profiles of CD11b and CD45 (Koeniger and Kuerten, 2017; Martin et al., 2017). Other, microglial valuable markers are *TMEM119* (Bennett et al., 2016; Satoh et al., 2016; Li et al., 2019), *P2RY12* (Mildner et al., 2017), *SALL1* (Buttgereit et al., 2016), and Siglec-H (Konishi et al., 2017) which distinguishes them from other macrophages. Studies suggested that *TMEM119* is expressed exclusively on Iba1+CD68^+^ microglia and not on infiltrated Iba1+CD68^+^ macrophages within demyelinating lesions of MS that govern the edge of active lesions in MS (Zrzavy et al., 2017), indicating that microglial activation is related to disease development while its expression is absent in immature microglia (Satoh et al., 2016). In experimental autoimmune encephalomyelitis (EAE) mice the appearance of inflammatory T cells in the CNS is consistent with the activation of CD11b + microglia (Murphy and LeVine, 2010; Wang et al., 2019). Microglial immune activation is present in MS lesions, and microglia play a role as phagocytes in demyelination, which is not well understood. Even though, cell membrane receptors present, at low levels than that observed in circulating antigen-presenting cells, still include those that are responsible for activation of T cells [MHC class I and II, CD40, and B7.1 and B7.2 adhesion molecules (LFA, CD11a, CD11b)], along with complement, cytokine, and chemokine receptors causing disease pathogenicity (Deng and Sriram, 2005). The pathogenesis of three neurodegenerative diseases discussed above involves majorly IBA1, CD11b*,* and TMEM119 molecules in microglial migration, motility and phagocytosis in response to disease pathogenesis.

## Pharmacological Basis and Strategies Targeting Microglia in Disease Conditions

Based on the microglia functions in regulating neuroinflammation, drug targeting key homeostatic proteins of microglia has been proposed as a potential approach to deal with inflammation in the brain which ultimately leads to neuronal damage. This can be achieved by regulation or modification of receptors present on microglia, signaling pathways like JAK/STAT and NF-κB, using drugs.

Receptors such as TLRs and CB2 has been reported as a novel pharmacological target for neurodegenerative disorders like PD. Candesartan cilexetil, is a synthetic, benzimidazole-derived angiotensin II receptor antagonist prodrug with antihypertensive activity also observed to inhibit TLR2 and TLR4 expression in PD which in turn may reverse microglial phenotype from pro-inflammatory to anti-inflammatory in the oligomeric α-synuclein microenvironment (Daniele et al., 2015; Liu et al., 2019). Another drug, rifampin, and its autoxidation product rifampicin quinone, an antibiotic regulates α-synuclein induced TLR2 and P2X7 dependent microglia inflammatory response under *in vitro* condition (Acuña et al., 2019). *In vitro* studies have shown that antagonist of TLR4 such as TAK-242 or RSLA reduces oxidative stress and microglia-mediated TNF-α production and neuronal death (Hughes et al., 2019).

JAK/STAT and NF-κB signaling pathways play important role in the polarization of microglia to the M1 state (Yan et al., 2018). Therefore, targeting this signaling pathway may provide an alternative strategy for the supression of the microglia-mediated neuroinflammation. Phytochemical from a-asarone from Annonaceae and Araceae species has been shown to inhibit the NF-κB signaling which suppresses microglia polarization to M1 state and reduce the production of pro-inflammatory cytokines in MPTP (1-methyl-4-phenyl-1,2,3,6-tetrahydropyridine) induced PD mouse model (Kim et al., 2015). Another drug, basically a bioactive flavonoid named Tanshinone I prevent nigrostriatal dopaminergic neurodegeneration by selectively inhibiting NF-κB signaling activation, and reverses microglia form M1 pro-inflammatory to M2 anti-inflammatory state (Wang et al., 2015b).

Apart from this, several drugs that are currently in use for a disease like lenalidomide for multiple myeloma, zonisamide for epilepsy, minocycline for infectious diseases, dimethyl fumarate for multiple sclerosis are capable of inhibiting activation of microglia and attenuating production of pro-inflammatory cytokines in a mouse model of PD (Liu et al., 2019). However, the mechanism of action of these drugs on microglia is still elusive.

CD70 derived adenosine A2A signaling, Histamine-4 receptor and NLRP3 mediated inflammasome in microglia has also been reported as a promising target in modulating microglial activation for PD (Rocha et al., 2016; Rajasundaram, 2018). Similarly, IL10, cyclic AMP, and vitamin D have been shown to enhance anti-inflammatory phenotype by converting microglia from M1 to M2 state in PD mice model (Subramaniam and Federoff, 2017). Intracerebral injection of IL-10 has been shown to decrease the expression of iNOS and increase the production of TGF-β in the MPTP mouse model (Schwenkgrub et al., 2013). Cyclic AMP in combination with IL-4 causes conversion of microglia from M1 to M2 state decreases the production of oxidative molecules and pro-inflammatory cytokines and improves the phagocytic activity in *in vitro* (Ghosh et al., 2016). Cyclic adenosine monophosphate (AMP) is a major microglia homeostasis regulator which in turn gets modulated by phosphodiesterase. PDE inhibitors such as rolipram, sildenafil, yonkenafil, and ibudilast have been proposed as a potential therapeutic drug for the neurodegenerative disorder, including MS (Liu et al., 2019). Vitamin D has been shown to inhibit microglial activation; a shift from M1 to M2 polarizes states which in turn protect dopaminergic neurons against neuroinflammation and oxidative stress in OHDA/MPTP induced mouse PD model (Calvello et al., 2017). Another molecular target PPARs has been shown to modulate microglial polarization. Pioglitazone, rosiglitazone is agonists of PPAR-g have shown anti-inflammatory and neuroprotective role (Omeragic et al., 2019). Kca3.1 inhibitors charybdotoxin, clotrimazole, senicapoc, and TRAM-34 reduce microglial neurotoxicity in AD pathology. Among them, Senicapoc has been used for the treatment of sickle cell anemia. Repurposing senicapoc has been shown to reduce the amyloid load, neuroinflammation, and improves neuronal plasticity in 5xFAD mice (Jin L. W. et al., 2019). Another pathway known as Kynurenine has been proposed to contribute to CNS disease by producing several s neuroprotective products during its metabolism. In microglia, an enzyme kyurenine 3-monooxygenase (KMO) expression converts KYN to neurotoxic metabolites such as 3-hydroxykynurenine (3-HK) and quinolinic acid (Quin). Studies have shown region-specific increased production of KYN as well as decreased KYM metabolism toward the production of neurotoxic microglia metabolites in post-mortem AD brain (Biber et al., 2019; Sorgdrager et al., 2019). However, understanding of the role of KYN metabolic products in the development and progression of AD, neurotoxicity, and its association with microglia-mediated neuroinflammation need to be addressed for identification of specific KYN metabolite inhibitor.

Pharmacological antibody-mediated targeting of TREM2 showed to activate TREM2 signaling which further increases microglial survival and migration (Zheng et al., 2017; Schlepckow et al., 2020). However, detail study about TREM2 protein normal function in microglia regulation and pathophysiology of the brain need to be addressed so that it can be investigated further for a promising drug targeting. S100A4 as a marker of microglial reactivity, suggesting the contribution of S100A4 regulated pathways to neuroinflammation, and identify niclosamide as a possible drug in the control and attenuation of reactive phenotypes of microglia, thus opening the way to further investigation for a new application in neurodegenerative conditions (Serrano et al., 2019). HuR, an RNA binding protein is a key regulator of many genes that make up the molecular signature of activated microglia, including increased production of proinflammatory cytokines, chemokines, and migration-associated factors. HuR may be a therapeutic target depending on the disease and stage of the disease. HuR expression is not limited to microglia, and thus it remains to be seen whether other cells could be adversely affected by its inhibition (Matsye et al., 2017). In the Intracerebral hemorrhage (ICH) brain, TLR4 is upregulated mainly in microglia (Lively and Schlichter, 2018). In the blood injection ICH model, deletion of TLR4 inhibits NF-κB signaling pathway and improves ICH outcomes (Lively and Schlichter, 2018). The *in vivo* and *in vitro* studies on glioma stem cells and BV-2 cells respectively showed that TLR4 activation is essential for M1 microglial polarization (Dzaye et al., 2016; Gong et al., 2016). Pinocembrin (5, 7-dihydroxyflavanone), a natural product extracted from Propolis, decreases M1 phenotype microglia, as evidenced by decreases in the number of the CD16/32 + microglia and iNOS expression in MACS-sorted CD11b + microglia. However, this effect of pinocembrin was abolished in TLR4lps-del mice with ICH, indicating that pinocembrin reduced microglial M1 polarization mainly by TLR4 inhibition (Lan et al., 2017). Cocaine self-administration also increases microglial activation. The current results also highlight a distinct molecular pathway in microglia through which cocaine increases BDNF, involving the phosphorylation of MeCP2 and subsequent translocation from the nucleus to the cytosol, which frees the BDNF promoter and permits its transcriptional activation. This leads to a significant release of BDNF by microglia (Cotto et al., 2018). Metformin, an anti-diabetic drug decreased Iba-1 staining in the dorsal horn and inhibits development of neuropathic pain in spared nerve injury (SNI) mice model. But the effect of metformin on microglia and in improving positive behavior was only observed in male mice indicating that the neuropathic pain modifying effects of metformin are sex-specific supporting a differential role for microglial activation in male and female mice (Inyang et al., 2019).

## Natural Products and Their Role in Combating Oxidative Stress and Their Effect on Microglia in the Regulation of Neuroinflammation

**FIGURE 2:**
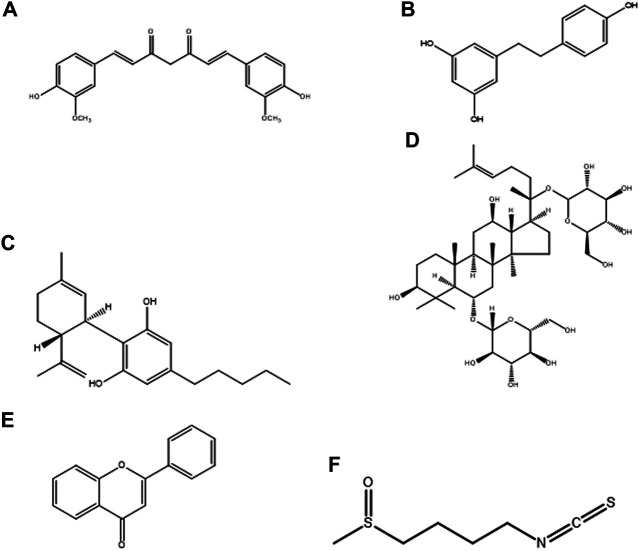
Chemical structure of natural products, **(A)** Curcumin, **(B)** Resveratrol, **(C)** Cannabidiol, **(D)** Ginsenosides, **(E)** Flavonoids, and **(F)** Sulforaphane.

Neurodegenerative diseases (NDs), mainly Alzheimer’s disease (AD), Parkinson’s disease (PD), amyotrophic lateral sclerosis (ALS), and multiple sclerosis (MS), onset and progression are characterized by oxidative stress, redox imbalance and neuroinflammation caused by microglia. The activated microglia is polarized into M1 (pro-inflammatory) and M2 (anti-inflammatory) functional phenotypes. One of the proposed therapeutic approaches for the treatment of NDs and related diseases is inhibition of the M1 phenotype while stimulating the M2 phenotype. Several natural products have been screened for their therapeutic modulatory role of microglial polarization in NDs (Jin X. et al., 2019). The major advantages of using these natural products are their novel structure (Figure 2), multi-target molecular mechanism, potent efficiency, and safe pharmacodynamics. In this section, we primarily summarized the therapeutic potential of natural products, such as curcumin, resveratrol, cannabidiol, ginsenosides, flavonoids, and sulforaphane, for their molecular mechanisms in the treatment of NDs by targeting microglial proteins.

### Curcumin

*Curcuma longa* L*.*, a member of the ginger family (Zingiberaceae), is a rhizomatous herbaceous perennial herb and is cultivated in countries with a tropical climate in Asia like India, China, etc. The rhizomes of turmeric (*Curcuma longa* L.) grow below the ground and curcumin is a major polyphenol isolated from them. *Curcuma longa* L. and curcumin has been used for thousands of years as a remedy and cited in the ancient Indian medical system, Ayurveda, for treatment of several illnesses, such as inflammation, gastric, hepatic, infectious diseases, and blood disorders. (Goel et al., 2008; Hatcher et al., 2008; Ghasemi et al., 2019). The phytochemical curcumin is a free radical scavenger and hydrogen donor and exhibits both pro-oxidant and antioxidant activity. In the experimental diabetic rats induced by streptozotocin (STZ), the effect of curcumin on liver anti-oxidative stress showed inhibition of polyol pathway associated with down-regulated expression of protein kinase C (PKC) and poly ADP ribose polymerase (PARP) in the curcumin-treated group compared with the diabetic model (Xie et al., 2017). Curcumin directly reacts and scavenge reactive oxygen species (such as superoxide anion, hydroxyl radicals, hydrogen peroxide, singlet oxygen, nitric oxide, peroxynitrite and peroxyl radicals). It further indirectly induces the expression of cytoprotective proteins as an antioxidant response, these includes superoxide dismutase (SOD), catalase (CAT), heme oxygenase 1 (HO-1), glutathione-S-transferase (GST), glutathione reductase (GR), glutathione peroxidase (GPx), NAD(P)H: quinone oxidoreductase 1 (NQO1) and γ-glutamylcysteine ligase (γGCL) (Trujillo et al., 2013). Recently the experimental model of the *Saccharomyces cerevisiae* budding yeast demonstrated that curcumin significantly increases oxidative stress and accelerates replicative and chronological aging of yeast cells devoid of anti-oxidative protection (with SOD1 and SOD2 gene deletion) and deprived of DNA repair mechanisms (RAD52). Curcuminoids decrease hepatic fat accumulation (hypertriglyceridemia) and avert accumulation of lipid droplets within hepatocytes (steatosis) by downregulating lipogenic factors and activating AMPK in liver (Sahebkar, 2014; He et al., 2017).

In brain, curcumin is a pleiotropic molecule showing therapeutic properties on microglia, such as inhibits microglia transformation, inflammatory mediators, and consequent NDs. CNB-001, a synthetic pyrazole derivative of curcumin, at a potent concentration (1–10 µM) suppressed the production of lipopolysaccharide (LPS)-induced nitric oxide (NO) and expression of inducible NO synthase (iNOS) in primary cultured rat microglia (Akaishi and Abe 2018). Further, this study suggested that the CNB-001 exerts its anti-inflammatory effects through inhibition of NF-κB and NO production by blockade of the *p*38 mitogen-activated protein kinase (MAPK) signaling pathways in microglia culture (Akaishi and Abe, 2018). Eun et al., 2017 investigated the neuroprotective effect of fermented *Curcuma longa* L (FCL) and its efficacy for the regulation of memory dysfunction in brain cell lines (C6, rat glioma cells, and BV2, murine microglia) and scopolamine-treated mice. The result indicated FCL inhibited the production of NO and prostaglandin E2 (PGE2) by the blockage of iNOS and cyclooxygenase-2 (COX-2) expression in BV2 cells. In the FCL, the curcuminoids content was 1.44%. (Eun et al., 2017). In another study, curcumin significantly inhibited LPS-mediated induction of COX-2 expression in both mRNA and protein levels in microglial cells. Additionally, curcumin distinctly inhibited LPS-induced NF-kB and activator protein-1 (AP-1) DNA bindings in BV2 microglial cells (Kang et al., 2004). Studies on AD pathology have shown numerous pharmacologic effects and mechanisms of action of curcumin, such as lessened β-amyloid plaques, deferred degradation of neurons, metal-chelation, anti-inflammatory, antioxidant, and reduced microglia formation, leading to the overall memory enhancement in patients with AD (Mishra and Palanivelu, 2008).

### Resveratrol

In recent years, a type of natural phenol resveratrol (RES) has fascinated scientists especially for its potent protective effect in NDs. The phenolic resveratrol acts as a commanding antioxidant in the presence of the ascorbate or glutathione system through both classical, hydroxyl-radical scavenging and a new, glutathione-sparing mechanism (Burkitt and Duncan, 2000). Resveratrol was found to act as an antioxidant, anti-inflammatory and anti-mutagen effects in leukemic HL-60 cells and acts on the major stages of carcinogenesis. It mediated anti-initiation activity by inducing phase II drug-metabolizing enzymes, anti-promotion activity by inhibiting cyclooxygenase and hydroperoxidase functions and antiprogression activity by inducing human promyelocytic leukemia cell differentiation (Jang et al., 1997). With regards to brain pathologies, the therapeutic potential of RES is through the modulation of microglial activation and regulation of neuroinflammation. The possible molecular mechanisms are direct antioxidant property of RES, TLR4 and NF-κB signaling pathway, SIRT1 and AMPK signaling, MAPKs signaling pathway, Nrf2/HO-1 signaling, and NLRP3 inflammasome signaling pathway (Huang et al., 2020). In the Yang et al., 2017 suggests RES reduces inflammatory damage and encouraged microglia polarization to the M2 phenotype via PGC-1α measured in *in vitro* and *in vivo*. Furthermore, they found RES ameliorated LPS-induced neuroinflammation behavior in mice models (Yang et al., 2017). It has been shown that the potential therapeutic activity of RES is by inhibition of COX-1 activity. Interestingly, RES potently reduced PGE2 synthesis and the formation of 8-iso-prostaglandin F2α (8-iso-PGF2α) in LPS-activated primary rat microglia. Resveratrol is reported as the first known inhibitor that specifically prevents the expression of mPGES-1 in a dose-dependent manner (mRNA and protein) without affecting the expression of COX-2 levels (Candelario-Jalil et al., 2007). mPGES-1 is a key enzyme for the synthesis of PGE2 by activated microglia cells.

### Cannabidiol

Cannabidiol (CBD), one of the important pharmacologically active Phytocannabinoids of *Cannabis sativa* L., is non-psychoactive, nevertheless has several valuable pharmacological effects including anti-inflammatory and anti-oxidant activity which are well documented (Iffland and Grotenhermen, 2017). In the keratinocytes isolated from the skin of psoriatic patients or healthy volunteers, the cannabidiol treatment has effect on the redox balance and phospholipid metabolism in UV-A/UV-B-irradiated keratinocytes (Jarocka-Karpowicz et al., 2020). The CBD treatment resulted in accumulation of cannabidiol in UV-irradiated psoriasis cells and keratinocyte membranes, lessened the oxidative phospholipid changes and increased the level of the level of palmitoylethanolamide (PEA) that leads to reduced inflammation (Jarocka-Karpowicz et al., 2020). In AD cases, CBD reduces oxidative stress, lessens mitochondrial dysfunction, and diminishes generation of reactive oxygen species. The CBD treated PC12 cells showed inhibition of amyloid beta (Aβ)-induced tau protein hyperphosphorylation linked to the downregulation of an inhibitor of Wnt pathway called as p-GSK-3β (glycogen synthase kinase-3β) (Vallée et al., 2017). They further showed CBD suppression is mainly through initiation of peroxisome proliferator-activated receptor γ (PPARγ), and pro-inflammatory signaling. In regards to oxidative stress management in brain, the rat model of mania showed therapeutic protection by CBD treatment in the brain damage against d-amphetamine-induced oxidative stress (Valvassori et al., 2011). While in human neuroblastoma cells (SHSY5YAPP+), CBD demonstrated neuroprotective properties by promoting ubiquitination of the amyloid precursor protein (APP), which is an indicator of cellular changes in the brain of patients with Alzheimer’s disease (Scuderi et al., 2014). Interestingly, increased endogenous cannabinoids or endocannabinoids (eCB) signaling is associated with an anti-inflammatory, neuroprotective phenotype in microglia (Araujo et al., 2019). The components of the endocannabinoid system (ECS) and signaling controls microglial activity include eCB (small, lipid-derived molecules), enzymes that metabolize these ligands, to cannabinoid receptor 1 (CB1) and cannabinoid receptor 2 (CB2). Microglial activity in CNS development and homeostasis can be modulated by eCB signaling, thus understanding the ECS can provide advanced therapeutics for autism spectrum disorder (ASD) and its associated conditions (Araujo et al., 2019).

### Ginsenosides

*Panax ginseng* (Genus: *Panax*; Family: *Araliaceae*) is a group of slowly growing plants with fleshy roots and Ginseng is the most valuable herbal medicine consumed globally (Tyler 1995). The unique triterpenoid saponins known as Ginsenosides are found exclusively in Panax species and have been isolated from roots, leaves, stems, fruits, and/or flower heads of ginseng. Ginsenoside Rd, a dammarane-type steroid glycoside, has showed encouraging neuroprotective efficacy and diminishes redox imbalance to improve stroke outcome in laboratory and clinical studies. Ginsenoside Rd has exhibited improved stroke outcome after focal cerebral ischemia in aged mice brain, attenuates redox imbalance and neuroprotection against ischemic cerebral damage both *in vitro* and *in vivo*. In 24 h post-ischemia, Ginsenoside Rd significantly repressed the build-ups of DNA, protein and lipid peroxidation products (Ye et al., 2011a). At 4 and 24 h after reperfusion, it showed to protect mitochondria as observed by preserved respiratory or electron chain complex activities and aconitase activity, hyperpolarized mitochondrial membrane potential and decreased hydrogen peroxide formation (Ye et al., 2011a). Ye et al., 2011b study showed in rats that the Ginsenoside Rd crossed the intact blood-brain barrier and employs neuroprotection in both transient and permanent middle cerebral artery occlusion (MCAO) (Ye et al., 2011b). Apart from this, Zhao et al., 2009 detected in hippocampus of 12 months old C57BL/6 mice, that many ginsenosides including Ra, Rb1, Rb2, Rc, Rd, Re, Rf, Rg1, Rg2, Rg3, and Ro were effective to prevent memory damage by upregulating plasticity-related proteins and modulation of synaptic plasticity. Some of the important ginsenosides-modulated CNS targets associated with potentiating of brain functions are Rg1 targets iNOS, NMDA receptor (of the hippocampus in ischemic gerbils), Synapses, L-type Ca2+ channels, P38 (modulation of COX-2 expression), DMT1 and FPI, IGF-1, GR CDK4, pRB, and E2F1; Rg3 targets voltage-dependent Ca2+ channels, glycine binding site of NMDA receptor, neprilysin gene, and MSRA; Rg2 targets α1β1δɛ or α3β4 subunits of NMDA receptor, V291, F292, and I295 residues of 5-HT (3A) receptor channels; Rb1 targets (cAMP)-dependent PKA, Choline acetyltransferase, NMDA receptor, α3β4 subunits of nicotinic receptors and P35; Rb2 targets transcription factor AP2 binding site; Rf targets unidentified G protein-coupled receptor; Rh2 targets Na+ channels, Polyamine binding site of NMDA receptor, PACAP and NF-κB and MAP kinase; Re targets Bcl2, Bax, iNOS. And caspase-3; Rd targets iNOS and COX-2, and GR; PNS targets ICAM-1 (Radad et al., 2011). The protopanaxadiol ginsenosides from *Panax ginseng* showed ginsenosides are positive modulators of P2X4 receptors. Further, *in silico* docking studies with a homology model of human P2X4 showed the possible ginsenoside binding site in the central vestibule region of the large ectodomain of P2X4 (Dhuna et al., 2019).

### Flavonoids

Flavonoids are a diverse group of plant metabolites or phytonutrients found in almost all fruits and vegetables. Flavonoids are thought to provide health benefits through their anti-oxidative, anti-inflammatory, anti-mutagenic and anti-carcinogenic properties. They are now considered a crucial constituent in a variety of nutraceutical, pharmaceutical, medicinal, and cosmetic products (Panche et al., 2016). The potential interventions of dietary flavonoids to improve redox imbalance in obesity and related co-morbidities (such as type 2 diabetes mellitus, CVD, gastrointestinal disorders and cognitive impairment) are well-researched and are also effective in reducing levels of oxidative stress and related inflammatory conditions (Gentile et al., 2018). The reduction of oxidative stress by flavonoids is coordinated through mechanisms regulated by the human body’s antioxidant system i.e., glyoxalase pathway, ability to scavenge ROS and regulate antioxidant defense mechanisms and cell signaling pathways (Frandsen and Narayanasamy, 2018). Fisetin, a plant flavonol, inhibited microglial cell migration, and ROS production in BV-2 microglial cells (Chuang et al., 2014). The regulatory molecular mechanism of fisetin-induced HO-1 expression works through the PI-3k/AKT and *p*38 signaling pathways in microglia cells. They also reported substantially reduced inflammation-related microglial activation and coordination deficit in *in vivo* mice model (Chuang et al., 2014). Several studies on flavonoids have shown that they exert their neuroprotection (Jang and Johnson, 2010) through impeding microglia activation and subsequent release of many inflammatory molecules such as Luteolin reduced IL-1β, TNFα, NO, PGE2 and attenuated neuronal cell death (Jang et al., 2010); Apigenin inhibited NO and PGE2 (Ha et al., 2008); Blueberry extract inhibited LPS-induced NO, TNFα and IL-1b in BV-2 experimental model (Lau et al., 2007). Isoflavone metabolites decreased LPS-induced NO and TNFα in primary microglia BV-2 (Park et al., 2007).

### Sulforaphane

Sulforaphane (SFN) is a potent activator of Nuclear Factor Erythroid 2-related factor 2 (Nrf2) and a natural dietary isothiocyanate, present in the species of the *Brassicaceae* such as broccoli and cabbage vegetables. In the cell, the master regulator of cellular detoxification responses and redox balance is known as Nuclear factor E2 factor-related factor (Nrf 2) which kindles the cellular defense mechanism and detoxification process. In our cellular redox system, sulforaphane treatment modulates redox status by activating redox regulator Nrf 2 (Ruhee and Suzuki, 2020). Further, reports suggest sulforaphane alters the cellular Keap1/Nrf2/ARE pathway that is useful for improving health and diseased conditions. Pyridoxamine (PM) and Sulforaphane (SFN) monotherapy in the Goto-kakizaki (GK) rats (an animal model of non-obese type 2 diabetes), triggered significant improvement in endothelial dysfunction in aorta and mesenteric arteries associated with type 2 diabetes, reducing vascular oxidative damage, advanced glycation end products (AGE) and HbA1c (hemoglobin A1c) levels (Pereira et al., 2017). SFN has potent anti-inflammatory activity and may have neuroprotective properties in diverse neurodegenerative diseases related to inflammation (Qin et al., 2018). The underlying mechanisms of SFN against microglia-mediated neuronal damage remain elusive. Qin et al., 2018 have shown that SFN significantly blocked the phosphorylation of both MAPKs (p38, JNK, and ERK1/2) and NF-κB p65, and with mitogen-activated protein kinase (MAPK) inhibitors (SB203580, SP 600125, and U0126) or an NF-κB inhibitor (PDTC) in BV-2 microglia cells. Their results on microglia-mediated neuronal necroptosis demonstrated that the SFN reduces LPS-induced pro-inflammatory processes through down-regulation of MAPK/NF-κB signaling pathway in BV-2 cells (Qin et al., 2018). Another study (Subedi et al., 2019) showed SFN exerts an anti-inflammatory effect on microglia by decreasing c-Jun N-terminal kinase (JNK) phosphorylation levels, subsequently causing a reduction in NF-κB and AP-1 signaling, and suppress the expression of the inflammatory mediators (iNOS, COX-2, NO, and PGE2) and proinflammatory cytokines (TNF-α, IL-6, and IL-1β). SFN also increased the expression of Nrf2 and HO-1 and the manufacture of the anti-inflammatory cytokines IL-10 and IL-4 (Subedi et al., 2019). Thus, the use of various natural products having anti-inflammatory and immunomodulatory effects has opened new avenues in the treatment of various neurological disorders.

## Molecular Docking of Iba1 With Curcumin, Resveratrol, Cannabidiol, Ginsenosides, Flavonoids, and Sulforaphane Indicates Iba1 as Promising Drug Target

**FIGURE 3:**
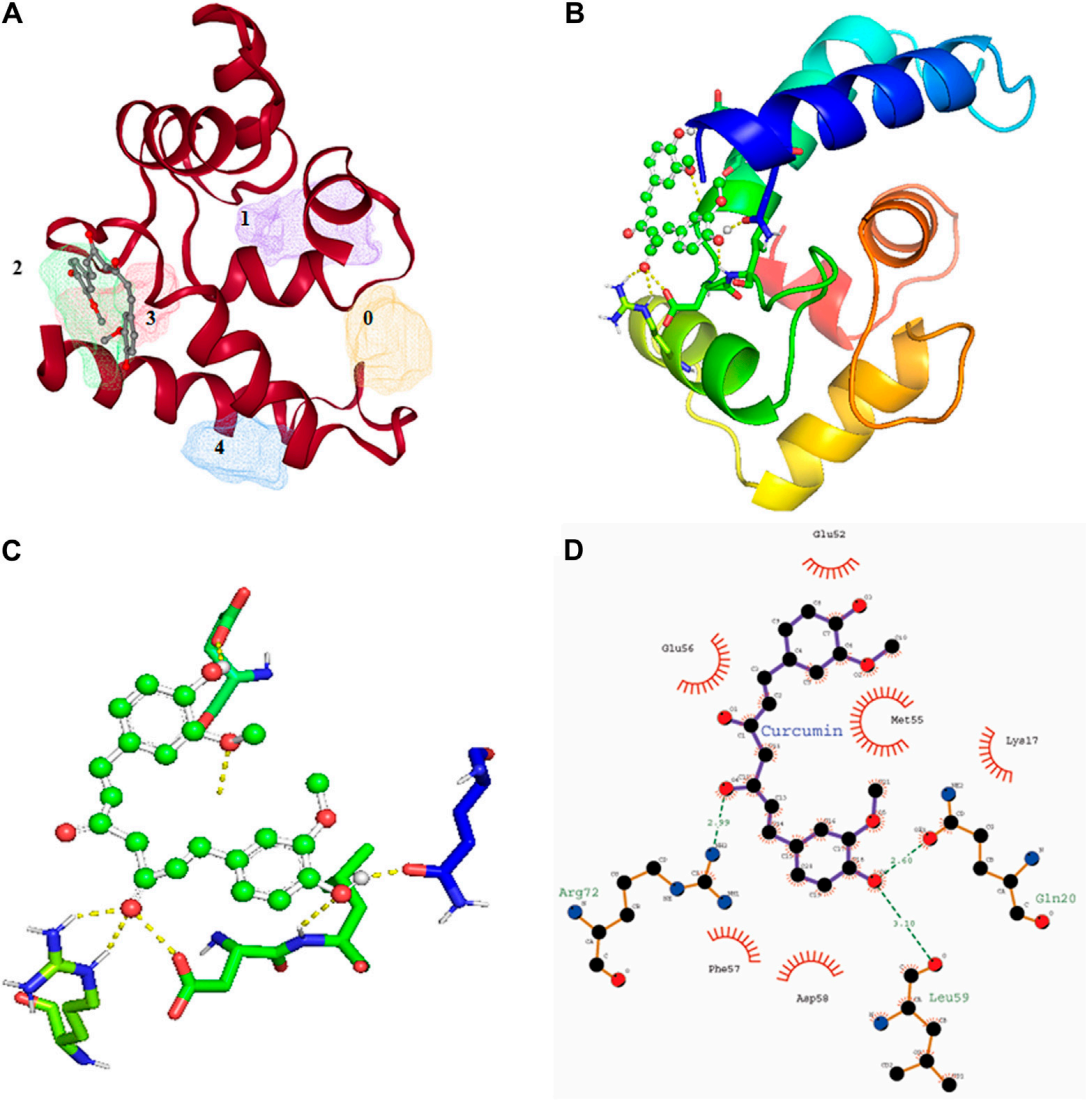
Molecular docking showing the interaction of Iba1 protein with Curcumin. **(A)** DoGSiteScorer analysis for predicting potential drug binding sites (pockets) on protein 3D structure. Out of 5 pockets for drug binding detected, Curcumin has been observed to fall in pocket 2 (green). **(B, C)** Pymol analysis of protein-ligand interaction after molecular docking using Autodock. The dotted yellow line indicates hydrogen bonding between Iba1 and Curcumin. **(D)** LigPlot Plus analysis of Autodock result generated 2D ligand-protein interaction diagram depicting hydrogen bonds between Iba1 Gln20, Leu59 and Arg72 amino acids with Curcumin. The hydrogen bond has been shown in green dotted line with a distance of protein and ligand hydrogen bond mentioned in Angstroms (Å).

Among all the know microglial genes, Iba1 has been consistently in use as a pan marker of microglia. It is a calcium binding protein, found in the cytoplasm of microglia and its expression has been observed upregulated in activated microglia. It regulated switching of microglia phenotypes during disease conditions. Iba1 participates in membrane ruffling and phagocytic activity of activated microglia via Rac signaling, by interacting with small G protein Rac. In microglia, Rac is a key molecule involved in proliferation, migration, regulation of actin cytoskeleton, microglia activation, phagocytosis and bioactive agents production. *In vitro* study suggest M-CSF, IL-6 and IFN-γ cause morphological changes to activated microglia and enhance Iba1 expression (Imai and Kohsaka, 2002). Recent study suggest that siRNA based silencing of Iba1 leads to reduced proliferation, cell adhesion whereas increased phagocytic activity of BV2 microglia cells indicating a new microglia target to modulate microglia and its functions (Gheorghe et al., 2020). Natural products are widely used in reducing redox imbalance and inflammation in different organ system including brain. However, molecular targets of these natural products are still not know. Therefore, we aimed to target microglia specific Iba1 protein for molecular docking studies using natural products. The human Iba1 3D structure was retrieved from Protein Data Bank (PDB ID: 2D58) and was used to perform molecular docking with natural products such as Curcumin, Cannabidiol, Ginsenosides, Resveratrol and Sulforane using AutoDock 4.2 (Morris et al., 2009). Further, visualization of drug pockets on Iba1 protein, protein-ligand interaction and analysis of hydrogen bond formation was analyzed by ProteinsPlus server (Schöning-Stierand et al., 2020) and LigPlot2. The DoGSiteScorer analysis of ProteinsPlus server for predicting potential drug binding sites (pockets) showed presence of 5 drug pocket sites on Iba1 which can be used for drug binding. The protein-ligand interaction using Curcumin as ligand showed its binding to drug pocket 2 of Iba1 with volume of 214.4 Å^3^ and surface area of 426.42 Å^2^ and drug score of 0.46 (Figure 3A; Table 1). Similar observation was also obtained for Cannabidiol, Ginsenosides, Resveratrol and Sulforane binding on Iba1 protein drug pockets. Further, visualization of Iba1 interaction with Curcumin showed 3 hydrogen bond between Gln20, Leu59 and Arg72 residue of Iba1 with Curcumin (Figures 3B,C) has a distance of 2.60 and 3.10 and 2.99 Å, respectively (Figure 3D) and binding energy of −4.79 Kcal/Mol and inhibition constant of value 306.23 µM (Table 1). Similarly, Cannabidiol, Ginsenosides and Resveratrol also showed lower binding energy with Iba1 with values of −4.84, −6.83 and −6.29 Kcal/Mol respectively, whereas number of hydrogen bonds between ligand and protein observed least in Ginsenosides as compared to Curcumin, Cannabidiol and Resveratrol (Table 2). Lower the binding energy between protein-ligand interactions, higher the affinity of ligand for the protein where presence of hydrogen bond and other hydrophobic interactions helps in forming good binding affinity (Azam and Abbasi, 2013). Drug targeting of any protein requires interaction between them. Our analysis for binding energy and interactions among selected natural products and Iba1 indicates promising therapeutic approach by targeting microglia specific genes in altered redox imbalance and neuroinflammation of brain during pathological conditions. Widespread screening of natural products for targeting microglia specific genes could be one of the approach in combating redox imbalance and neuroinflammation in brain pathologies. However, further analysis of microglia specific proteins like Tmem119, Siglec H, CD40 etc. with natural products would help us in identifying more details on promising drug target for microglia.

**TABLE 1:** Binding site prediction granularity: Properties and druggability for Iba1.

S. No	Name of pocket	Volume (Å³)	Surface area (Å^2^)	Drug score
1	P_0	249.98	514.11	0.38
2	P_1	214.4	426.42	0.46
3	P_2	172.29	328.74	0.34
4	P_3	161.79	262.52	0.27
5	P_4	121.54	305.22	0.22

**TABLE 2:** Protein-ligand interaction analysis of Iba1 with natural compounds showed the lowest binding energy with Ginsenosides and Resveratrol.

S.No	Protein	Compound	RMSD	Binding energy (Kcal/Mol)	Inhibition constant (Ki) (µM)	No of H bonds (drug-enzyme)	Amino acid involved in the interaction
1	Iba1	Curcumin	53.495	−4.79	306.23	3	Gln20, Leu59, Arg72
2	Iba1	Cannabidiol	241.650	−4.84	281.83	3	Arg23, Asp64
3	Iba1	Ginsenosides	22.641	−6.83	9.38	1	Lys17
4	Iba1	Resveratrol	58.577	−6.29	24.55	3	Glu92, Val93, Arg114
5	Iba1	Sulforane	18.741	−3.36	3.45	1	Arg114A

## Conclusion

Redox imbalance in microglia plays a critical role in neuroinflammation and neurodegeneration, where several promising druggable targets are being identified in recent studies. Molecular docking results from microglia specific proteins and diverse range of natural products like Curcumin, Cannabidiol, and Resveratrol support targeting microglia using natural products as a possible intervention to regulate redox imbalance and inflammation in neurological diseases. Precise drug designing and *in vivo* studies are warranted to further validate the drug molecules in mitigating the microglia induced neurodegeneration implicated in several neurodegenerative diseases.
